# Pellucid-like keratoconus

**DOI:** 10.12688/f1000research.1-48.v1

**Published:** 2012-11-15

**Authors:** Mazen M Sinjab, Lara N Youssef

**Affiliations:** 1 Damascus University, Damascus, Syria

## Abstract

**Purpose: **To study the tomographic features of pellucid-like keratoconus (PLK), and to report a new sign on the pachymetry map (PM) in pellucid marginal degeneration (PMD).

**Patients and methods:** A retrospective descriptive case series was performed in Damascus University in 2011. Clinical and tomographic findings of 15 eyes (9 patients) that had the claw pattern of the anterior sagital map (ASM) were reviewed. Patients were distributed into two groups: (1) 4 eyes were considered PMD since they had inferior corneal thinning on both slitlamp biomicroscopy and PM; (2) 11 eyes were considered as PLK since they did not show inferior corneal thinning. Patients were studied using slitlamp biomicroscopy and Scheimpflug-based tomography (Pentacam HR). The ASM, anterior elevation map (AEM) and PM were analyzed and compared to study the “kissing birds” sign, the “bell” sign, and cone location.

**Results:** Patients’ average age was 25.93±8.05 (16–44 years). In total, 60% of patients were male. In group 1, the AEM in the best fit sphere (BFS) mode revealed no kissing birds sign, and the cone was central in 1 eye (25%) and paracentral in 3 eyes (75%). PM showed the bell sign in 4 eyes (100%). In group 2, the AEM in the BFS mode revealed the kissing birds sign in 2 eyes (18.2%), and the cone was central in 1 eye (9.1%), paracentral in 8 eyes (72.7%) and peripheral in 2 eyes (18.2%). PM didn’t show the bell sign in any eye.

**Conclusion: **The claw pattern on the ASM is not a hallmark of PMD; it can be seen in PLK. Cone location does not relate to diagnosis. The “bell” sign on the PM is a deferential diagnostic sign in PMD.

## Introduction

Pellucid marginal degeneration (PMD) is an idiopathic, progressive, non-inflammatory, ectatic corneal disorder characterized by a peripheral inferior band of corneal thinning in a crescent-shaped pattern ^1^, although PMD cases with areas of superior thinning have been reported ^2^.

Similarities between PMD and keratoconus (KC) have led some ophthalmologists to consider PMD to be a peripheral form of KC ^2, 3^. Distinguishing between the two entities is of potential clinical importance since they differ markedly in prognosis and management. The management of PMD is unique since PMD is a progressive disease despite the fact that it is encountered in the third to fifth decade of life. Accordingly, corneal cross linking should still be one of the treatment options. When intracorneal rings (ICRs) implantation is indicated in the management of PMD, caution should be paid to the location of the inferior segment, since it passes through the inferior thinned area. Hence the need to calculate the depth of implantation depending on the thinnest point on the resumed passage, rather than on the thickness of the site of incision, in order to avoid deep corneal penetration.

**Figure 1.**
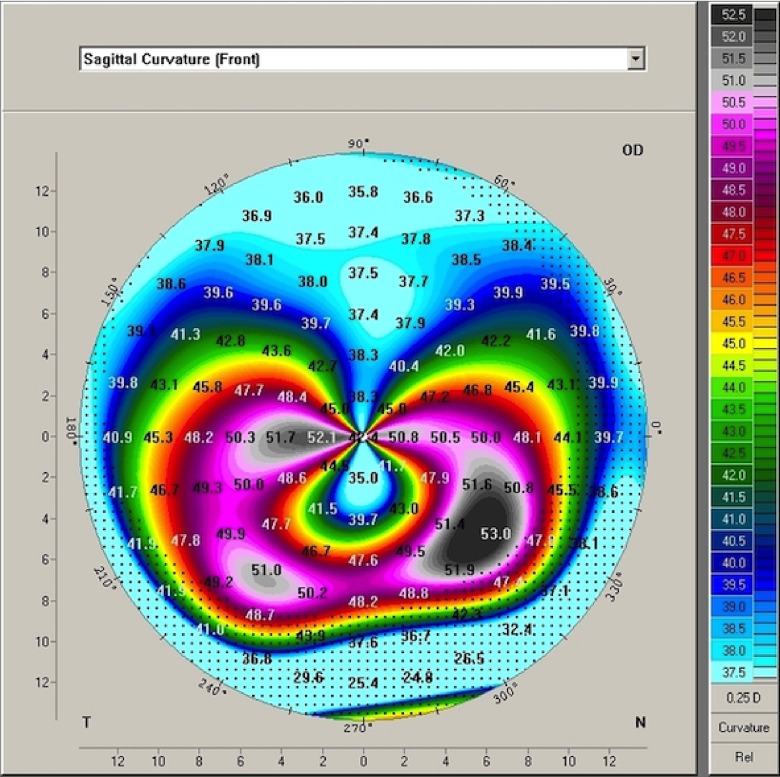
The crab claw or butterfly pattern on the anterior sagital curvature map. Notice the marked flattening of the cornea along the vertical meridian and the marked steepening of the inferior corneal periphery, which extends into the mid-peripheral inferior oblique corneal meridians associated with against-the-rule astigmatism.

In PMD, corneal tomographic analysis reveals a flattening in the vertical meridian, inducing a significant against-the-rule (ATR) astigmatism and a significant steepening around the area of maximum thinning ^3^. This corneal configuration corresponds to a tomographic map that shows the classical claw pattern (Figure 1).

Although corneal tomography is an important tool for the diagnosis of this corneal pathology, it should not be used as the only diagnostic criterion because it has been shown that this pattern is not always associated with the diagnosis of PMD; it might be seen with some other corneal ectatic disorders ^4^. Therefore, pachymetric and biomicroscopic findings must also be considered for a reliable diagnosis.

In our study, we are reporting a "bell-shaped" sign on the pachymetry map (PM) in PMD which corresponds to the inferior thinning of the cornea observed by the slitlamp biomicroscopy. We are also reporting cases of KC with claw pattern on the sagital map but with neither the bell sign on the PM nor the inferior thinning on slitlamp biomicroscopy, identifying these cases to be “pellucid-like keratoconus (PLK).”

## Patients and methods

A retrospective descriptive case series was performed in Damascus University in 2011. Clinical and tomographic findings of 15 eyes (9 patients) were reviewed and qualitatively analyzed. Inclusion criteria consisted of having a classic crab claw pattern on the anterior sagital curvature map (see Figure 1) taken by Pentacam® HR corneal tomographer (OCULUS Optikgeräte GmbH, Germany).

**Figure 2.**
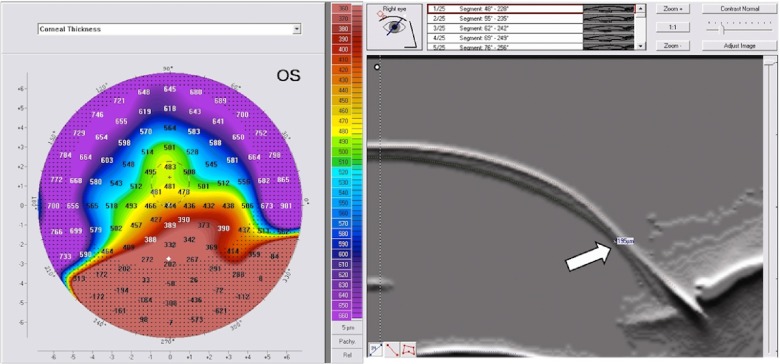
Inferior corneal thinning on corneal pachymetry in PMD. A: Bell sign on the thickness map, it is an indicator of inferior corneal thinning; compare values between the inferior part of this cornea with other peripheral parts. B: Scheimpflug image showing the thinning (white arrow).

**Figure 3.**
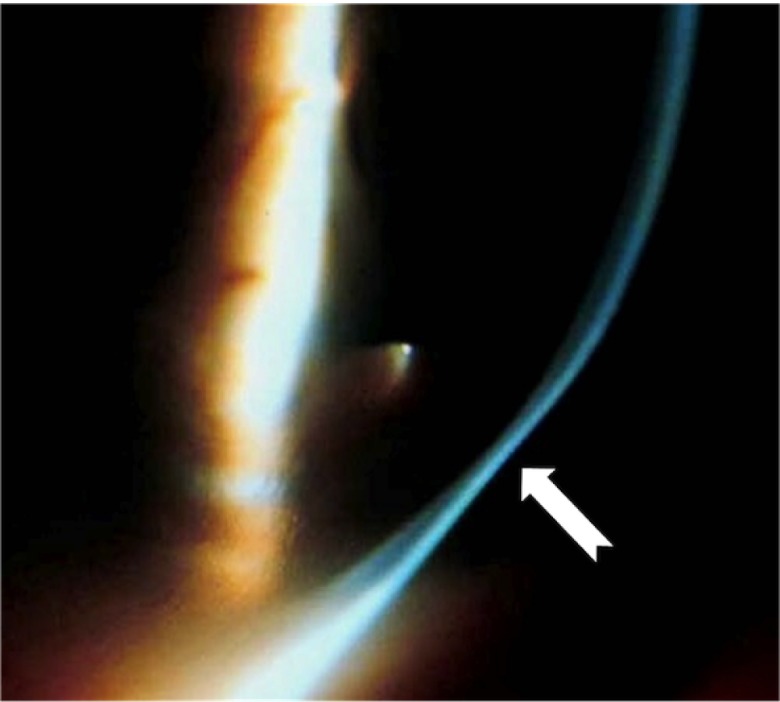
Inferior corneal thinning on Slitlamp biomicroscopy in PMD. Notice the abrupt narrowing of the slit beam in the area of inferior thinning (white arrow).

Patients were distributed into two groups based on clinical and tomographic findings. The diagnosis of PMD was based on both corneal tomography and clinical findings of peripheral corneal thinning in an arcuate or crescentic pattern on slitlamp biomicroscopy ( Figure 2 and Figure 3). Therefore, group 1 was considered PMD; group 2 was given the name of “pellucid-like keratoconus (PLK)” since the inferior thinning was not observed in this group. The PMD group (group 1) consisted of 4 eyes that had the claw pattern on the anterior sagital map (ASM), and an inferior corneal thinning on slitlamp biomicroscopy. The PLK group (group 2) consisted of 11 eyes that had the claw pattern on the ASM, but without an inferior corneal thinning on slitlamp biomicroscopy.

**Figure 4.**
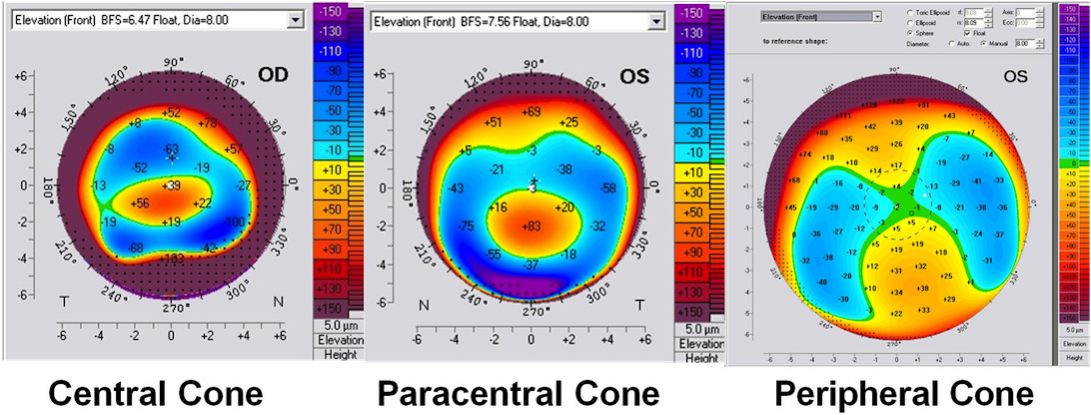
Classification of cone location on the anterior elevation map with the BFS float mode. The cone is central when its apex is within the 3mm central circle, paracentral when it is in between 3 and 5mm central circles, and peripheral when it is out of the 5mm central circle.

Anterior sagital curvature, anterior elevation, and pachymetry maps were qualitatively analyzed and compared between groups 1 and 2. The anterior elevation map was studied using the best fit sphere (BFS) float mode to localize the cone and to identify the kissing birds sign. The cone was considered central, paracentral or peripheral when the apex of the cone was within the central 3 mm zone, within 3–5 mm zone or out of the central 5 mm zone respectively (Figure 4).

## Results

Patients’ average age was 25.93±8.05 (16–44) years. A total of 60% of the patients were male. Average age of patients was 23±7.31 (16–33) years in group 1, and 27±8.03 (19–44) years in group 2.

Table 1 summarizes the results in both groups regarding the crab claw pattern, kissing birds sign, cone location, bell sign and inferior thinning on slitlamp biomicroscopy. In group 1, the cone was paracentral or central in 75% or 25% of cases respectively. Claw pattern, bell sign and inferior thinning were found in 100% of cases. In group 2, the kissing bird sign was present in 18.2% of cases, the cone was paracentral, peripheral or central in 72.7%, 18.2% or 9.1% of cases respectively. Bell sign and inferior thinning were absent.

**Table 1.** Clinical and topographical findings in groups 1 and 2.

		**Crab claw** **(anterior sagital map)**	**Kissing birds** **(anterior elevation map with** **BFS float mode)**	**Cone location** **(anterior elevation map with BFS float mode)**			**Bell sign** **(Thickness map)**	**Inferior** **thinning** **on slitlamp**
				**Central**	**Paracentral**	**Peripheral**		
Group 1: 4 eyes (PMD)	No. of eyes	4	0	1	3	0	4	4
	%	100	0	25	75	0	100	100
Group 2: 11 eyes (PLK)	No. of eyes	11	2	1	8	2	0	0
	%	100	18.2	9.1	72.7	18.2	0	0

## Case report

A 27–year-old woman presented to the outpatient department with the complaint of blurred vision in both eyes. The uncorrected distance visual acuity (UDVA) was 0.7 (decimal) in the right eye (OD) and 0.2 (decimal) in the left eye (OS). On examination, the manifest refraction was -1.75D Cyl @65° OD, and-6.00D Cyl @105° OS. Corrected distance visual acuity (CDVA) was 1.0 (decimal) OD, and 0.7 (decimal) OS. Slitlamp biomicroscopy of the right eye revealed a clear cornea and normal features. The left eye showed inferior peripheral band of thinning extending from the 4 o’clock position to the 8 o’clock position. The intraocular pressure was normal in both eyes. Fundus examination revealed normal features. There were neither systemic nor local associations.

**Figure 5a and b.**
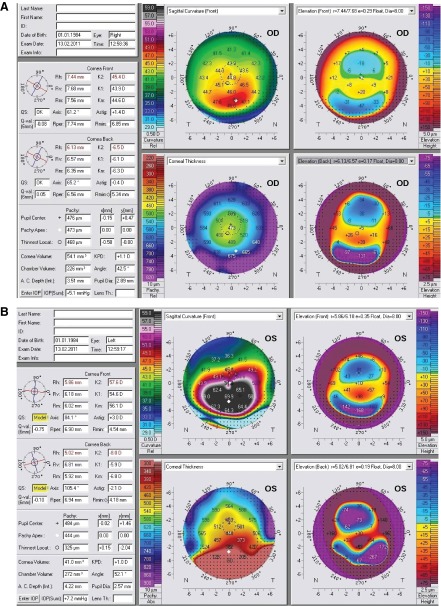
A case with PMD in one eye and PLK in the other. ( **A**): The right eye is PLK; notice the absence of the bell sign. ( **B**): The left eye is PMD; notice the presence of this sign.

Corneal tomography (Figures 5a and b) revealed inferior peri-limbal steepening with the crab claw pattern on the anterior sagital curvature map in both eyes. The anterior elevation map with BFS float mode revealed paracentral cones without the kissing birds sign in both eyes, the PM showed that the bell sign was present in the left eye and absent in the right eye. Therefore, diagnosis of PLK OD and PMD OS was made.

## Discussion

In addition to careful clinical examination, corneal tomography has been recognized as an important and sensitive tool in detecting and managing ectatic corneal disorders, such as PMD and KC ^5, 6^. According to some reports, the typical sagital curvature map of the condition shows marked flattening of the cornea along a vertical axis and a steepening of the inferior cornea peripheral to the site of the lesion ^3^. On the other hand, Lee BW et al. reported cases of ectatic corneal disorders with the same pattern ^4^. Therefore, careful studying of corneal tomography with the main three maps is mandatory for diagnosing PMD and differentiating it from other ectatic corneal disorders. The main three maps consist of the anterior sagital curvature map, anterior elevation map and PM.

In our study, the crab claw pattern on the ASM was the inclusion criteria. Thereafter, cases were divided according to the presence or absence of the inferior corneal thinning on slitlamp biomicroscopy. Group 1 was identified as PMD when the thinning was present, and group 2 was identified as PLK when this sign was absent. We studied the other main maps qualitatively in both groups.

**Figure 6.**
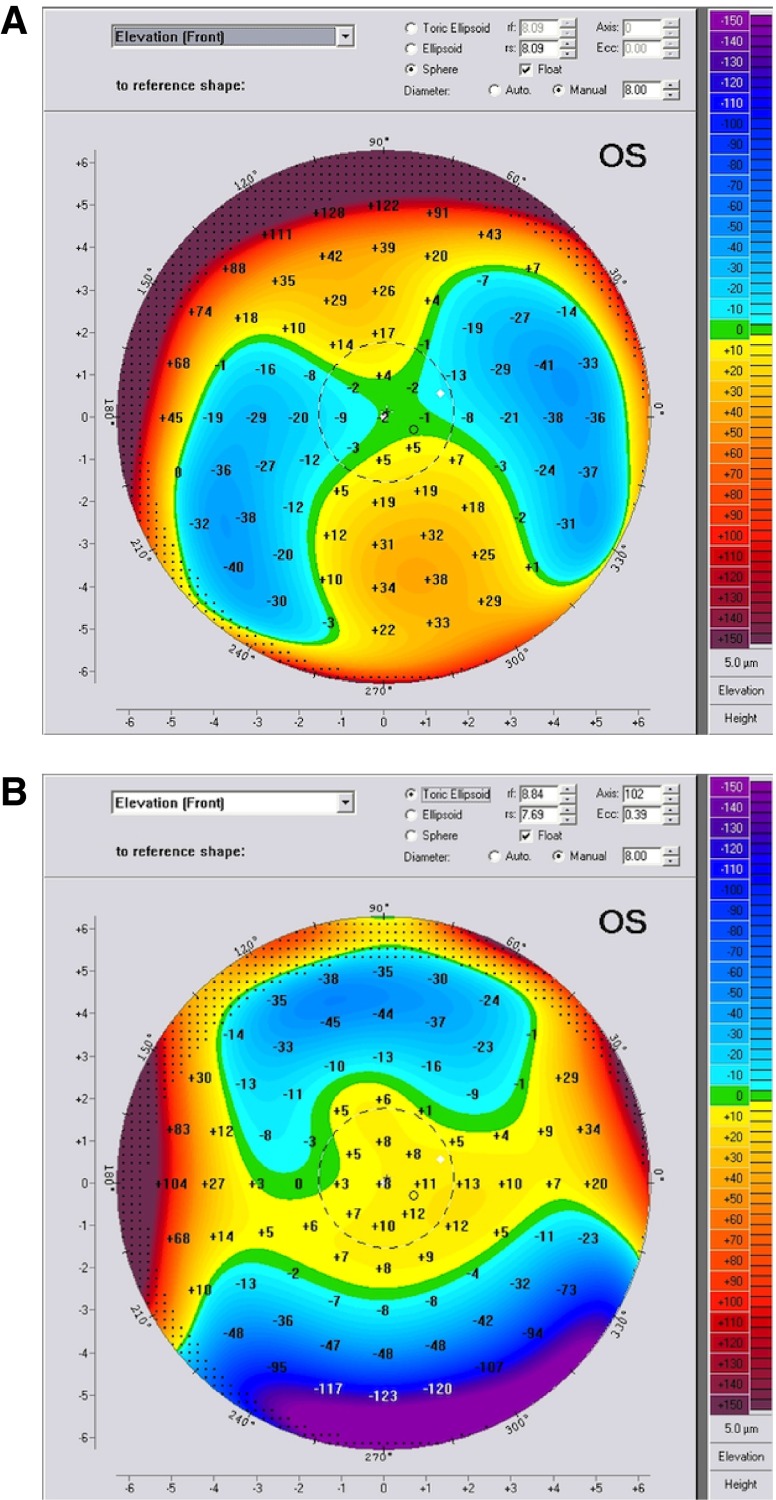
The kissing birds sign on the anterior elevation map. When it exists, it appears on the BFS float mode ( **A**), and disappears when switching to the BFTE mode ( **B**).

On the anterior elevation map, the kissing birds sign can be seen, and theoretically it should be always seen in PMD since it is a peripheral ectatic corneal disorder. In our study, we found that the presence of this sign was related to cone location. When the cone was peripheral, this sign was present; when the cone was central or paracentral, this sign was absent This sign appeared when the BFS float mode was used, while it disappeared when switching to the best fit toric ellipsoid (BFTE) float mode, as shown in Figure 6. In group 1, the cone was central or paracentral in 100% of cases, and the kissing birds sign was absent in 100% of cases. In group 2, the cone was peripheral in 18.2% of cases, in which the kissing birds sign was present.

**Figure 7.**
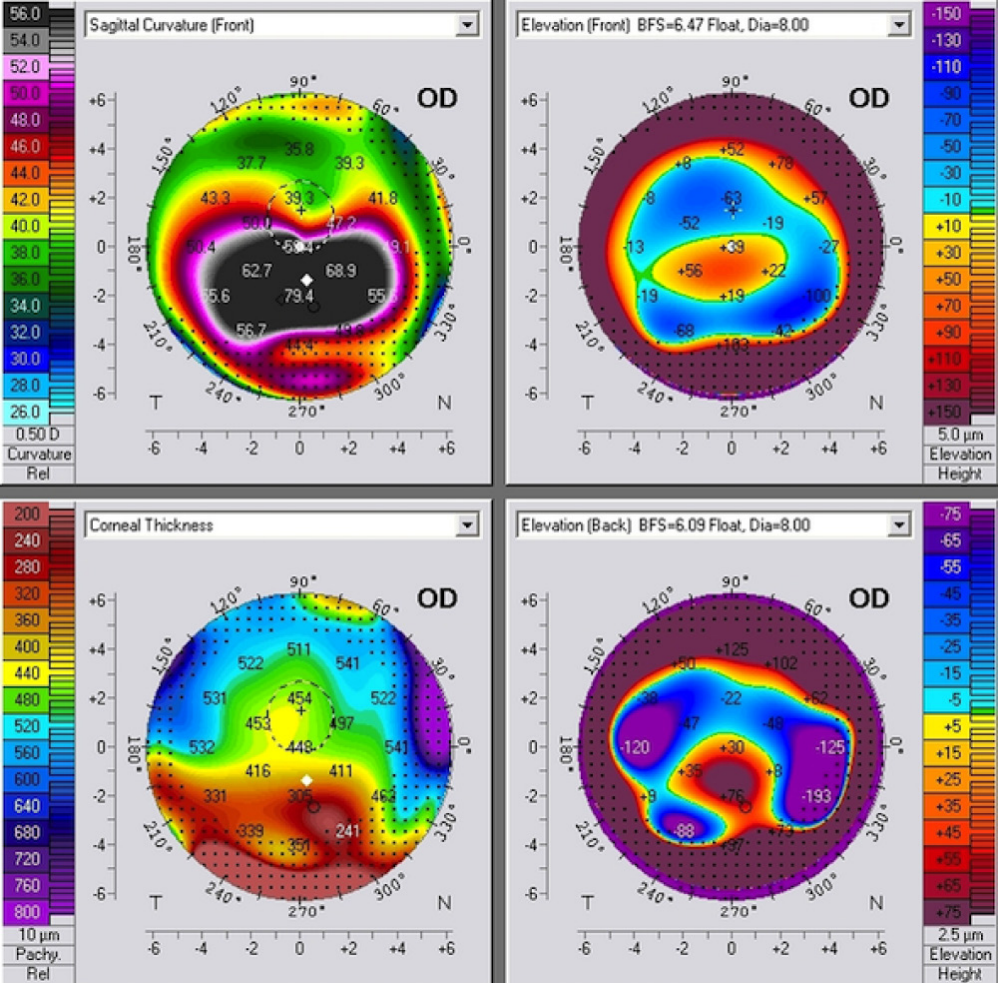
A PMD case without the kissing birds sign. Notice the bell sign on the corneal thickness map.

Cone location can be identified on the elevation maps or on the tangential curvature map, but not on the sagital curvature map ^7^. In our study, we found no correlation between cone location and the differentiation between the two groups; i.e. the cone could be central, paracentral or peripheral in both entities. Therefore, neither the kissing birds sign nor the peripheral cone was a hallmark of PMD. Figure 7 is a PMD case without the kissing birds sign.

The PM showed thinning in PMD corneas towards the inferior part of the cornea, which was consistent with what was observed by the slitlamp biomicroscopy; a peripheral band of thinning of the inferior cornea from the 4 o'clock position to the 8 o'clock position and the light slit became very narrow abruptly in the inferior part of the cornea, which was the hallmark of the disease. This thinning was characterized with a special sign on the PM that can be called "bell" sign as shown in Figure 2. This sign was present in 100% of cases in group 1 and was absent in 100% of cases in group 2. Therefore, the hallmark of PMD was the bell sign on the PM, and this sign was due to inferior corneal thinning encountered in PMD.

On the other hand, there was no correlation between cone location and the thinnest area of the ectatic cornea. In group 1, although the bell sign was present and was an indicator of inferior thinning, the cone was central or paracentral in 100% of cases. On the other hand, the bell sign was absent in 100% of cases in group 2 although there were 2 cases (18.2%) with peripheral inferior cones.

**Figure 8.**
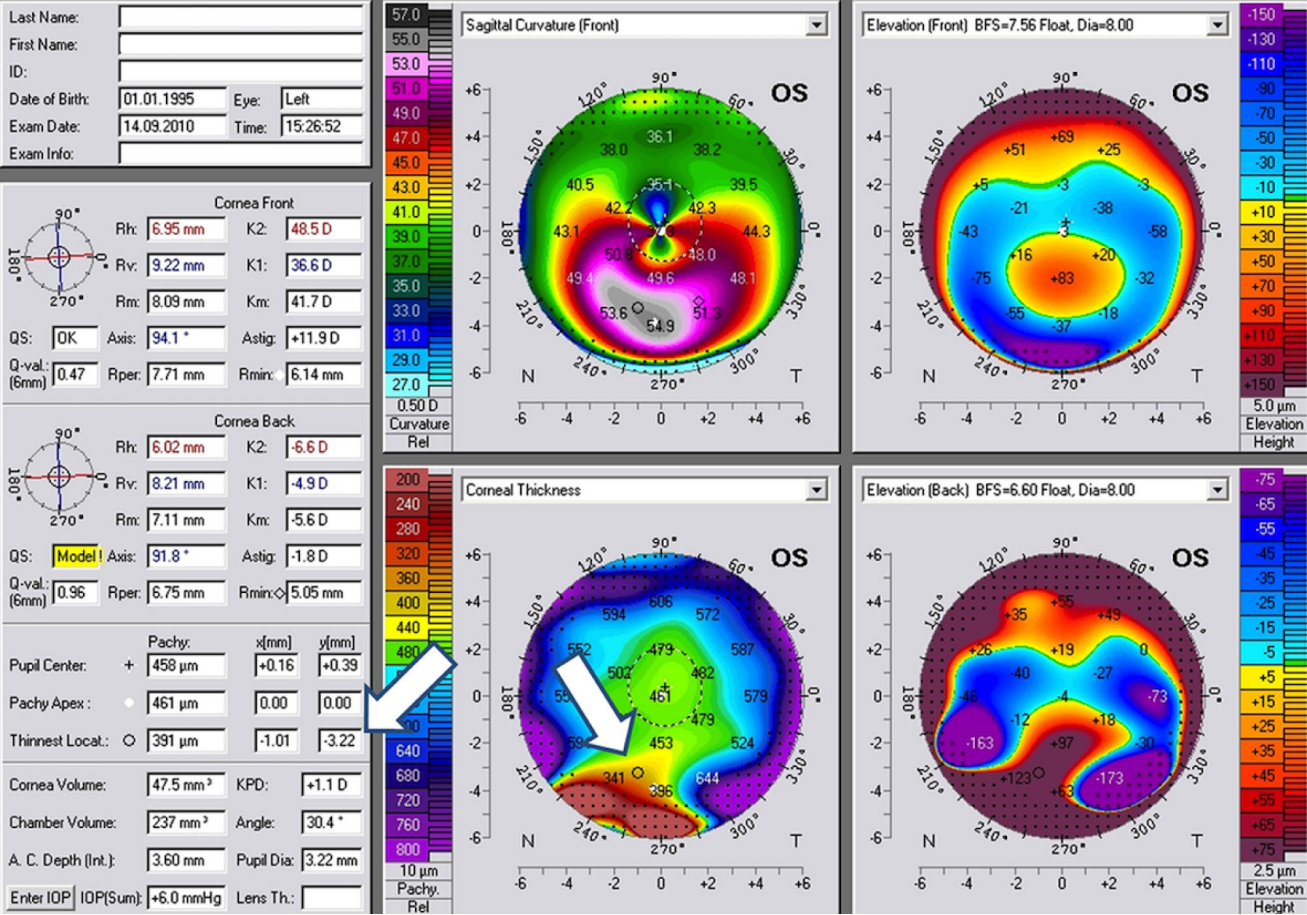
A case of severe PMD. The thinnest location is largely inferiorly displaced (white arrows).

**Figure 9.**
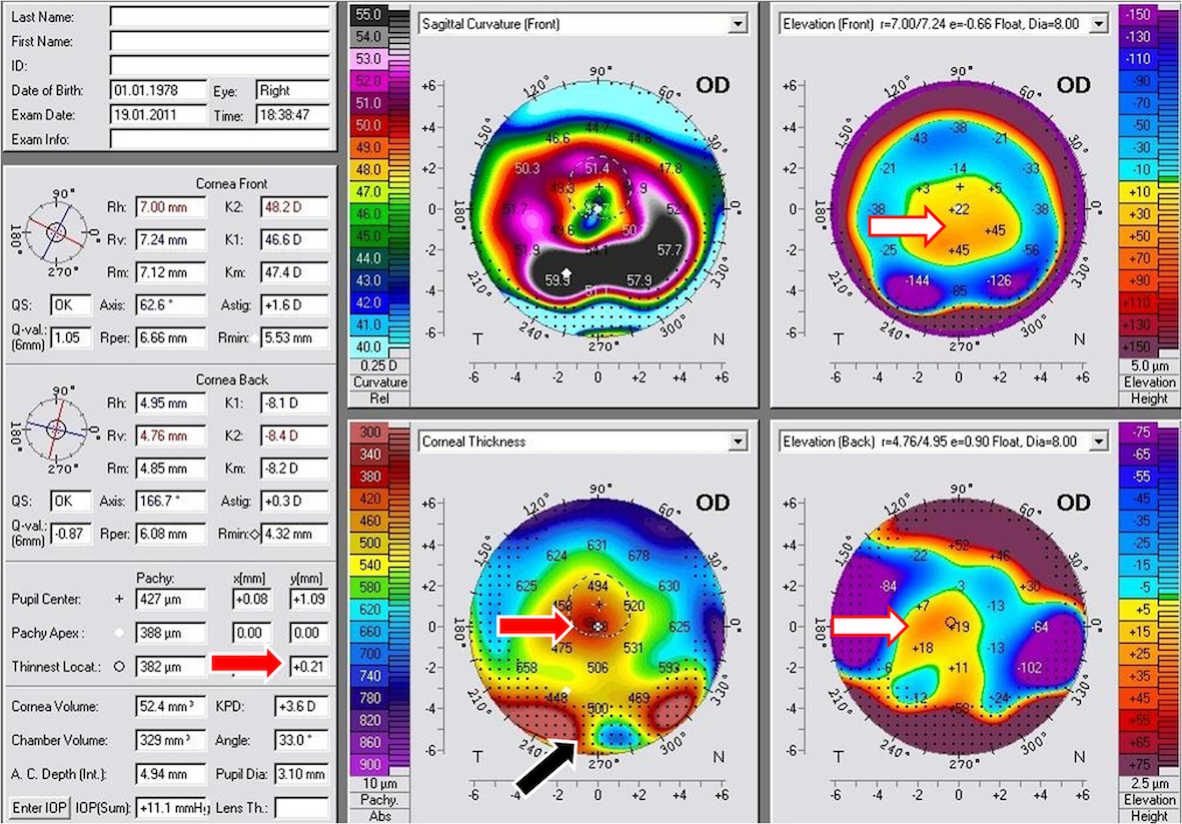
Corneal tomography of a severe PMD case with extrapolated data. The red arrows point to the thinnest location. The white arrows point to cone location. Black arrow points to a black dotted area suggesting that some data were missing in the inferior cornea due to severe corneal distortion.

In regard to the location of the thinnest point of the cornea, theoretically, the thinnest point should be inferiorly displaced in both PMD and in KC, but the amount of displacement on the *Y* coordinate should be much larger in PMD, especially in advanced cases ( Figure 8: white arrows). This is not always true. In very advanced PMD, the cornea is severely distorted, particularly in the inferior part of the cornea, and the tomographers may miss many data from this part, and extrapolate the area with dark dots indicating that an important part of the data was missing in this area, as shown in Figure 9.

It is well known in the literature that PMD usually starts later in life than KC: it presents in the third to fifth decade of life ^3, 5^. In our study, the mean age of presentation was the third decade in both PMD and PLK groups. There was one patient with bilateral PMD and his age was 16 years at presentation. In a series of 58 patients reported by Sridhar and Mahesh ^8^, there was an 8–year-old patient with PMD. Young ages were also seen in group 2; the age of the patients ranged from 19 to 44 years. Thus, patients' age is not an important issue when differentiating PMD from KC.

PMD and KC are bilateral ectatic corneal disorders, although they can be asymmetric in severity between both eyes ^8^. Nevertheless, some studies reported the occurrence of PMD in one eye and KC in the fellow eye ^9^. In our study, we reported a case with PMD in one eye and PLK in the fellow eye.

Regarding the case report, taking into account the clinical features, high ATR astigmatism, typical corneal tomography features and the presence of the bell sign on PM supported by peripheral corneal thinning, a diagnosis of PMD was made in the left eye and PLK in the right eye.

In conclusion, we reported cases of KC mimicking the tomographical appearance of PMD, and we called that pellucid-like keratoconus (PLK). Moreover, identifying features of PMD on corneal tomography and slitlamp biomicroscopy is very important since there are some tomographical similarities between PMD and PLK, especially in early stages of the former which misguides doctors to misinterpret PLK as PMD. We also reported a sign on the PM in PMD, and we called it the “bell” sign.

Finally, the similarity between the two entities, in addition to what we found in the reported patient who has both entities, may suggest that PLK could represent a closely related disorder to PMD, just as Forme Fruste KC is to KC.
