# Supplementary material for: Pellucid-like keratoconus
Source: F1000Res. 2012 Nov 15;1:48. [Version 1] doi: 10.12688/f1000research.1-48.v1 (PMC3752625; doi:10.12688/f1000research.1-48.v1)
Supplement: Peer review history

# Peer review: Referee response for version 1

**Published:** 2012-11-27

**Authors:** Natalie Afshari

**Affiliations:** Department of Ophthalmology, Duke University School of Medicine, Durham, NC, USA

The authors of the case report introduce the idea of pellucid like keratoconus (KC). They report cases of KC with a claw pattern on the sagital map but with neither the bell sign on the Pellucid nor the inferior thinning on the slit-lamp biomicroscopy. This idea is new and therefore worthy of publication.

I have read this submission. I believe that I have an appropriate level of expertise to confirm that it is of an acceptable scientific standard.

# Response

**Published:** 2012-11-27

**Authors:** Mazen Sinjab

**Affiliations:** Damascus University , Syria

Dear Professor Afshari,

During my past 16 years of practice as a refractive surgeon, I have seen more than 3000 cases of keratoconus and ectatic corneal disorders at my center and in the refractive clinic in the Damascus University hospital, and many of those cases were referred to me for a second opinion. This number seems to be large, but the cause behind it was that in Syria such diseases are relatively common due to close intermarriage, environment and to the high prevalence of vernal keratoconjunctivitis; this registers an incidence higher than the international one which is 1:2000, to be about 1:400 in Syria, and in some well known regions in Syria one can say 1:100. PMD consists of about 20% of the seen cases, and it is seen not only in adults but also in young patients, which gives the disease a more aggressive nature. The same can be said about KC, I have seen many cases with ages younger than 10 years old (the youngest was 4 years old!). Due to such a high number of cases that I have seen, I could recognize cases that lie in between KC and PMD although both entities have their own tomographic features; and that was the idea behind my study.

# Peer review: Referee response for version 1

**Published:** 2012-11-26

**Authors:** Michael W Belin

**Affiliations:** Department of Ophthalmology, University of Arizona, Tucson, AZ, USA

The authors appear not to be aware of the current literature which clearly distinguishes the tomographic patterns of inferior keratoconus from Pellucid. The limitation of curvature analysis and the importance of the pachymetric map have been previously well documented. The authors reference list is outdated and lacks articles that clearly describe the tomographic findings of true Pellucid: Walker RN *et al.* (2008), Belin MW *et al.* (2011) and Belin MW *et al.* (2012).

The anterior curvature patters described as “crab claw” or many other descriptors are non-specific and highlight the limitations of curvature when trying to imply shape. Adding additional descriptors (e.g. bell shape) is counterproductive.

The authors use the term “tomographic” without making a distinction between anterior curvature (classic topography) and tomography which includes both anterior and posterior corneal elevation and corneal thickness maps.

The pachymetric “bell sign” is also a poor descriptor and is seen in this example because of the scale chosen. If the authors had chosen an appropriate scale that covered the full range of thickness, they would see the more classic inferior band that has been previously described and published.

The case report is fraught with mistakes. First the authors keep stating that the best-fit-sphere was used but the machine is set for a toric-ellipsoid. The pachymetric map has not reliable data inferior, which is why no thickness readings are produced. One cannot diagnose true PMD in this case.

All in all, the paper is poorly researched and the maps show a lack of understanding of the displays.

I have read this submission. I believe that I have an appropriate level of expertise to state that I do not consider it to be of an acceptable scientific standard, for reasons outlined above.

# Response

**Published:** 2012-12-12

**Authors:** Mazen Sinjab

**Affiliations:** Damascus University , Syria

Dear Prof Belin, Thank you so much for your valuable comments.

Before I make any changes in my article, I would like to ask some questions. I have read the article “ Scheimpflug Photographic Diagnosis of PellucidMarginal Degeneration” by Walker RN *et al.* (2008), I will refer to it as article number 1; and the article “ What’s in a name: Keratoconus, Pellucid Marginal Degeneration and Related Thinning Disorders” by Belin MW *et al.* (2011), I will refer to it as article number 2. Unfortunately I could not have the third article which is “Simplified Nomenclature for Describing Keratoconus” by Belin MW *et al.* (2012). I would be very thankful if you could provide me with it and it would be so kind of you.

The questions are:

**1.** In article 2: the author stated: “in our original review article PMD was described as a typically bilateral, clear, inferior, peripheral corneal thinning disorder, characterized by a narrow band of corneal thinning (often approaching 20% normal thickness) separated from the inferior limbus by a relatively uninvolved area 1 to 2 mm in width.” However, nothing was mentioned in this article and in article 1 about the new definition of PMD and whether it is bilateral or can be unilateral. So the question is: can PMD be unilateral?

**2.** In article 2 the author stated: “Ertan and Bahadir in 2006 described the use of the femtosecond laser and intrastromal rings to correct PMD. While no corneal thickness maps were presented, the posterior elevation maps were typical of KCN and not classic PMD.” But may I ask: what is typical of KCN and not classic PMD on the posterior elevation map? The same question about what the author mentioned about Kubaloglu and associates.

**3. **How do you define that the cone is central, paracentral or peripheral on the elevation map with the BFS mode? I mean when do say the the cone is central (within 3 mm, 4mm, 5mm….) since this was not mentioned in both articles?

**4.**In article 2, the author recommended that “extrapolated data should not be displayed” and thus the 9mm scale should be used, therefore according to this recommendation, the peripheral part of the cornea will not be displayed or estimated and the pachymetric changes of PMD will not be evaluated?! At the same time, the authors use the extrapolated areas in many of their talks during conferences and articles!

**5.** In article 1: how could the authors recognize the thinning in case of focal edema? And how could they depend on Placido or even Scheimpflug in this very distorted cornea? They also stated:” a bilateral superior flattening” in figure 4 but only OS was displayed; What about OD, is it possible to see the flattening on OD despite edema even if it was focal and inferior?

**6.** In article 1, page 2: the author stated:” thinning within 2 mm of the limbus, and a more normal corneal thickness inferior to the band of thinning.” But all of that lie in the extrapolated area which should not be displayed as the author recommended in article 2!

Sincerely Yours, Mazen

# Response

**Published:** 2012-12-13

**Authors:** Michael Belin

**Affiliations:** Universtiy of Arizona, USA

Dr. Sinjab, I sent you the requested article via this publisher.

In reference to the findings of KCN vs PMD on the posterior elevation, Inf KCN shows the classic positive islands of elevation when using the standard BFS (sphere). Elevation maps are not overly useful in true PMD because the cornea is so flat in the central 8.0 mm zone that the reference surface is so flat as to not accentuate the pathology. This is why the pach map is the most important (opened up to show the full cornea)

As far as which is a central cone, vs paracentral, vs peripheral. I do not believe there is anything established but I use the central 3.0 for “central” and outside 6.0 for “inferior” but it is often just a visual description.

In general we limit our display to 9.0 mm as for routine screening this is easier. That is not the case for peripheral disease. In cases of true PMD, you have to show the entire cornea. It is true that the more peripheral the data the more likely it will be extrapolated but if the Pentacam produced a thickness reading it is useful for visual diagnosis. If the number is produced, but extrapolated, it means it did not meet the strict machine criteria. If no number (thickness) is produced then the map needs to be repeated as there is no useful data.

In article 1 the thinning was easily seen in the other eye as this was bilateral. The Scheimplfug images were still reliable in the eye with the hydrops except for the far periphery so the central and paracentral flattening could still be seen. The reason both eyes were not shown in all displays is a limit of the journal for case reports.

# Response

**Published:** 2012-12-13

**Authors:** Mazen Sinjab

**Affiliations:** Damascus University , Syria

Thank you very much Prof. Belin, and many thank to this journal that gave me the opportunity to learn from you. You were so kind to send me the article.

But may I ask you: could PMD be unilateral?

Sincerely Yours, Mazen

# Response

**Published:** 2012-12-14

**Authors:** Michael Belin

**Affiliations:** Universtiy of Arizona, USA

UNILATERAL – I don’t know. I would guess that if PMD (as is KCN) is ultimately a disorder of collagen (i.e. genetic / biochemical) that the collagen would be abnormal in both eyes though clinical presentations may be unilateral or highly asymmetic.

# Peer review: Referee response for version 1

**Published:** 2012-11-19

**Authors:** Virender Sangwan

**Affiliations:** L V Prasad Eye Institute, Kallam Anji Reddy Campus, Hyderabad. Andhra Pradesh, India

This is a well done study with a very good documentation of the case.

I have read this submission. I believe that I have an appropriate level of expertise to confirm that it is of an acceptable scientific standard.

# Response

**Published:** 2012-11-19

**Authors:** Mazen Sinjab

**Affiliations:** Damascus University , Syria

Thank you Virender for your endorsement.
